# Comparison of Shaped Charge Jet Performance Generated by Machined and Additively Manufactured CuSn10 Liners

**DOI:** 10.3390/ma14237149

**Published:** 2021-11-24

**Authors:** Shengjie Sun, Jianwei Jiang, Shuyou Wang, Jianbing Men, Mei Li, Yawei Wang

**Affiliations:** State Key Laboratory of Explosion Science and Technology, Beijing Institute of Technology, Beijing 100081, China; bitssj@bit.edu.cn (S.S.); bitjjw@bit.edu.cn (J.J.); menjb@bit.edu.cn (J.M.); pulmmay@bit.edu.cn (M.L.); 3120200247@bit.edu.cn (Y.W.)

**Keywords:** selective laser melting, CuSn10, shaped charge liner, jet performance, penetration capability, microstructures

## Abstract

The Selective Laser Melting (SLM) technique has attracted attention in a wide range of manufacturing research areas, including the defense industry because of its high efficiency and good consistency of manufactured material properties. Shaped charge liner is the crucial unit in the shaped charge warhead. However, jet performance formed by SLM-produced liner remains to be studied systematically. In the present research work, the SLM technique was applied to manufacture CuSn10 shaped charge liners. Casted CuSn10 liners were also fabricated using the classical turning method for comparison. The grain size of the SLM-produced liner was found to be much smaller than the machined liner due to the rapid heating and cooling rate during the SLM manufacturing process. This contributed to improved jet performance. A flash X-ray photography system was applied to capture jet stretching appearances. Necking appears at the jet tip formed by the machined liner, while the jet formed by the SLM-produced liner remains continuous. Penetration test results show that the penetration depth of the jet formed by the SLM-produced liner is around 27% larger than that formed by the machined liner. Segments along the sidewall of the penetration tunnels were selected for in-depth micro analysis. Energy dispersed spectrum (EDS) surface scanning results indicate the composition at the side wall of the penetrated tunnel. Metallurgical microscope was applied to distinguish four different phase zones of the target. The width of these different zones indicates the severity of the lateral interaction between the jet and target, which can be adopted to evaluate jet penetration capability. The present study analyzes the factors that influence jet performances and proves that SLM technology is well-adapted in the manufacturing of shaped charge liners.

## 1. Introduction

Shaped charge liner (SCL) is the crucial unit in the shaped charge warhead, which is widely accepted as the default device to create severe damage on thick armor targets. The liner material turns into jet and slug after converging at the charge axis driven by the explosive production of the main charge [1]. Before interacting with the target, the jet stretches to a length of 4–5 times the charge diameter due to the velocity and temperature gradients existing along the jet. Dynamic recrystallization under the high strain rate and high temperature conditions is considered to be the dominant factor that affects jet stretching stability [2,3].

During the jet penetration process, the pressure of the interaction area reaches 1~2 orders of magnitude of the target strength with the absolute temperature rising to a few thousand Kelvin and a high strain rate area is formed [4]. Part of the energy is transferred along the penetration direction to increase penetration depth. After the jet velocity reduces to a threshold value, the remaining energy expands laterally to increase the diameter of the penetration trajectory [5]. According to Birkhoff et al., the penetration depth of a shaped charge jet into a target is proportional to ${\left(\rho_{j}/\rho_{t}\right)}^{1/2}$, where P is the penetration depth, $\rho_{j}$ is the jet density, and $\rho_{t}$ is the target density [6]. However, apart from the densities of the jet and the target, extra factors can also influence jet penetration.

Murr et al. observed the liner grain size before and after explosion loading. They found that different liner material has a different grain size ratio [7,8,9]. A relationship between volumetric energy (*E*_0_) stored in the material and the starting grain size (*D*_0_) was proposed, and they believe that the volumetric stored energy is the critical issue in controlling shaped charge jet stability [10], which results in the influence of penetration capability. Huang et al. applied equal channel angular pressing (ECAP) technology to produce shaped charge liners with a grain size of 0.5–3.0 μm. Comparison of the penetration results revealed that the shaped charge jet formed by liners with ultra-fine grain outperforms the coarse-grain liner in terms of penetration [11,12]. These studies imply that a smaller liner grain size is beneficial in improving jet performance and the methods of refining grain size during the die forging process have been studied [13].

Nowadays, additive manufacturing (AM) has become a revolutionary technology across multiple industries [14]. Selective laser melting (SLM) is a representative fusion-based method to obtain three-dimensional structured devices [15], which is practical in controlling the grain size of the fabricated material due to the high cooling rate associated with the AM process [16]. H. O. Agu et al. compared the starting grain size and the ending grain size of machined liner and 3D printed liner. A numerical simulation method was applied to illustrate the mechanism of grain size changing [17]. Several investigations show that the penetration performances of the SLM fabricated liners are equal to or even better than the conventionally machined liners [18,19,20], which reflects satisfactory adaptability of the SLM in fabricating SCLs.

The penetration depth of a shaped charge jet is the dominant criterion for evaluating jet performance. Studies on the microstructure of penetrated targets have illustrated the mechanism of jets interacting with targets by observing the representative zone of target materials [21,22]. However, few studies have compared the jet performance of SLM-produced liners and machined liners and further details remain to be discovered.

In the present work, CuSn10 SCLs were fabricated using SLM technology and the classical turning method, respectively. The microstructures of these two liners were observed using metallographic microscope. Jet forming tests and penetration test were implemented. The flash X-ray photography system was used to capture jet appearances. Retrieved targets were cut along the axis of the penetration tunnel to evaluate the penetration capability. Segments along the tunnel sidewall were selected for micro-analysis, including metallurgical and SEM observation, EDS surface scanning and Vickers micro hardness measurement. Based on the relevant results, the factors that influence the jet forming performance and the penetration capability were analyzed.

## 2. Material Preparation

### 2.1. SLM Fabrication Process

The powder used in the SLM fabrication was made from the comminuted metals through gas atomization technology and the mixed particle diameter lies between 15μm and 53 μm. The composition of the powder is listed in Table 1.

**Figure 1:**
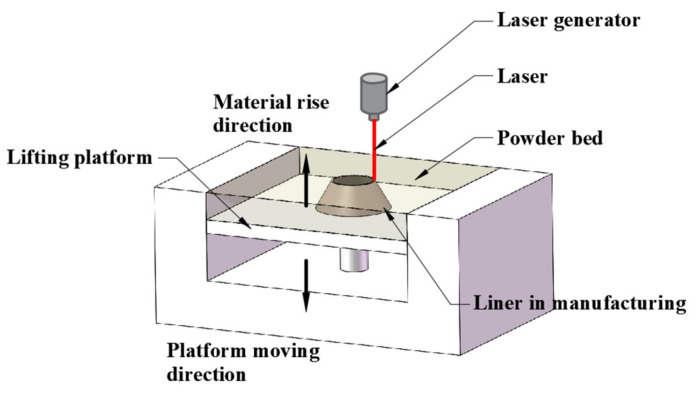
Schematic diagram of SLM manufacturing a liner.

**Figure 2:**
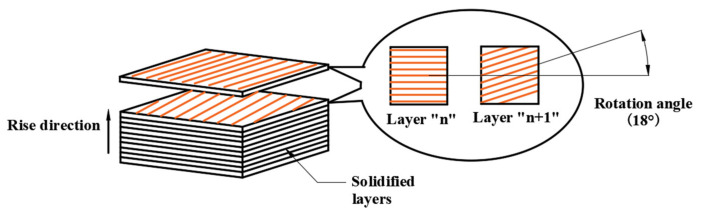
Layered structure produced by SLM technique.

The powder was directly applied to the SLM process, which was accomplished by the fiber laser with 1070 nm wavelength. The laser spot size on the surface of the powder bed was about 100 μm. Argon gas was used as the protective gas during the manufacturing process to prevent oxidation. The power of the laser used was 290 W, with a scanning speed of 1000 mm/s. The SLM manufacturing process is illustrated in Figure 1. The specific procedures are: (a) a thin layer metal powder is spread on a lifting platform; (b) laser beam selectively melts the powder and a solidified layer is shaped; (c) the lifting platform moves one layer thickness lower, which is 20 μm in the present study, and next layer of powder is spread; (d) the laser scanning direction is rotated 18° before melting the next layer of powder, which leads to the same rotation angle of the deposition stripes, as illustrated in Figure 2; (e) a new layer is solidified based on the preceding one. By repeating the mentioned procedures, liners were built layer by layer along the rise direction. After the SLM fabrication work, the produced liners were heat treated at 600 °C for 1 h in a N_2_ protective atmosphere to relieve the residual stress and trigger grain recrystallization. The effect of the heat treatment on SLM-produced material properties has been discussed in [23], which shows that the mechanical behavior of the SLM-produced materials can be tuned within a wide range of strength and ductility through proper annealing treatment.

### 2.2. Liners

Liners used in the present study were fabricated using the SLM technique and classical turning method. The powder and fabrication process of the SLM technique has been introduced in Section 2.1, based on which, the liners were produced.

**Figure 3:**
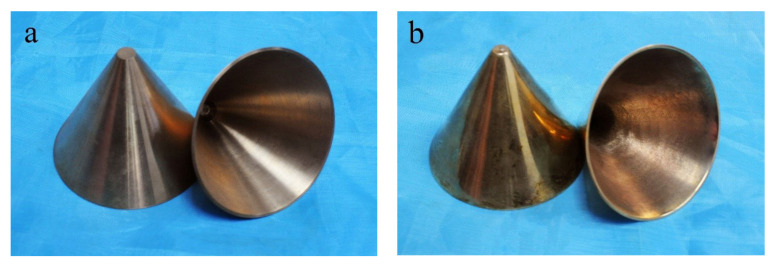
(**a**) Machined liners and (**b**) SLM manufactured liners.

CuSn10 rods with a diameter of 60mm were casted according to ISO 197-4:1983 standard [24]. Then, the casted rods were fabricated into the liners using the turning method. The geometry of the turned liner and SLM-produced liner is completely the same. Figure 3a,b show the machined liners and the SLM manufactured liners, respectively.

### 2.3. Shaped Charges

**Figure 4:**
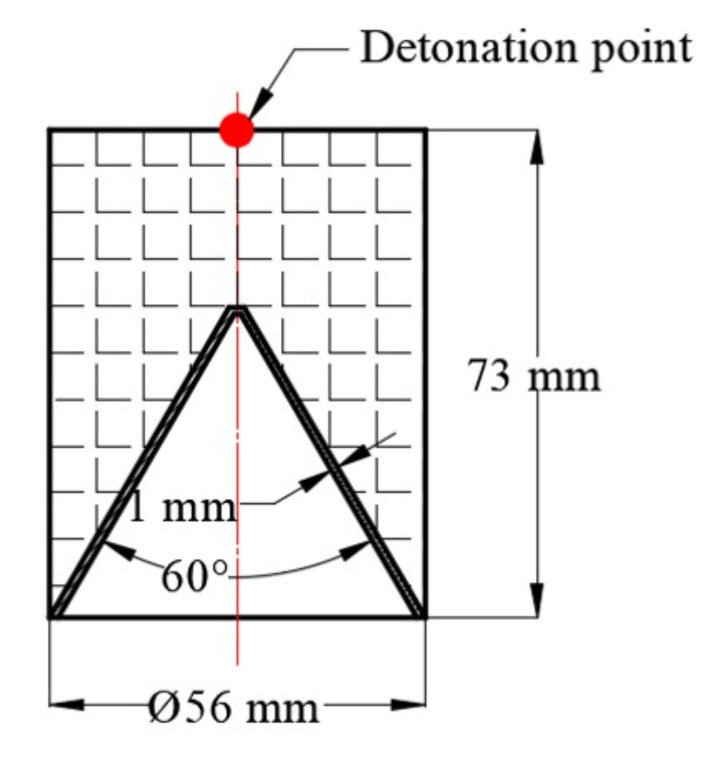
Sectional diagram of the shaped charge.

In order to compare the jet performance generated by the machined liner and the SLM-produced liner, a standard shaped charge design was applied to shape the liner into the jet. The main charge used in the present work is pressed JH-2, the composition of which is 95 wt% cyclo-1,3,5-trimethylene-2,4,6-trinitramine (RDX), 3 wt% C7H6N2O4 (DNT) and 2 wt% polyvinylacetate (CZ). The density of JH-2 is 1.70 g/cm^3^ and the detonation speed is around 8km. Details of the basic mechanical properties of the explosive was recorded in reference [25]. The sectional diagram of the shaped charge is shown in Figure 4.

**Figure 5:**
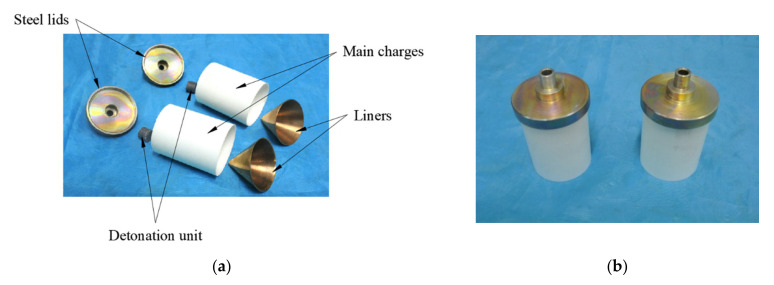
(**a**) Components of the warhead and (**b**) integrated warheads.

A detonation unit was adopted to trigger steady detonation propagated inside the main charge and a steel lid was adopted for assembly use. The components of the shaped charge warhead are shown in Figure 5a. Each of the components were assembled using shellac adhesive. Figure 5b shows the integrated warhead.

## 3. Experimental Design

### 3.1. Observation of Liner Microstructure

**Figure 6:**
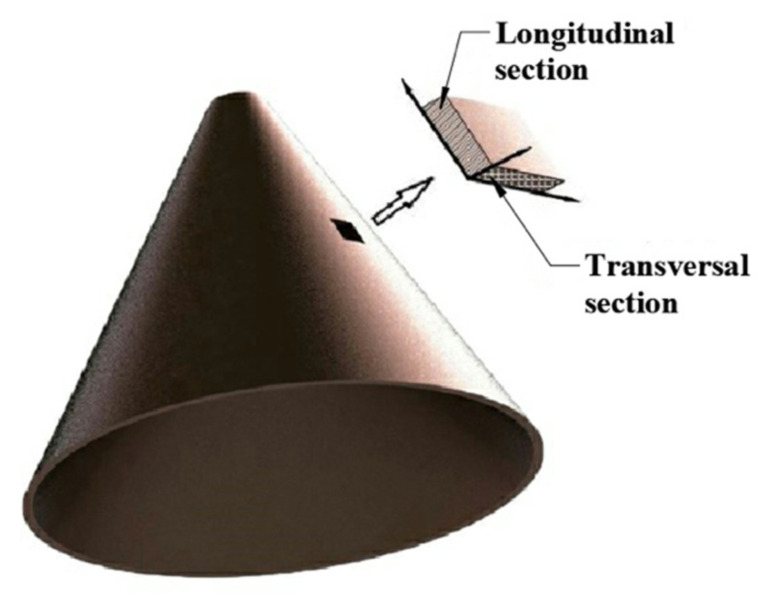
Longitudinal and transversal sections of the segments cut out from a liner.

In order to compare the micro view of the liners fabricated by different techniques, two segments were cut out from each of the liners and embedded into epoxy resin to make samples for observation. The geometry of the selected segment is 10 mm × 10 mm × 1 mm. Both the longitudinal section and transversal section, shown in Figure 6, were polished and etched with a solution composed of 100 mL water and 35 mL nitric acid. A micro view of the sections were observed using MV3000 metallographic microscope.

### 3.2. Jet Performance Tests

**Figure 7:**
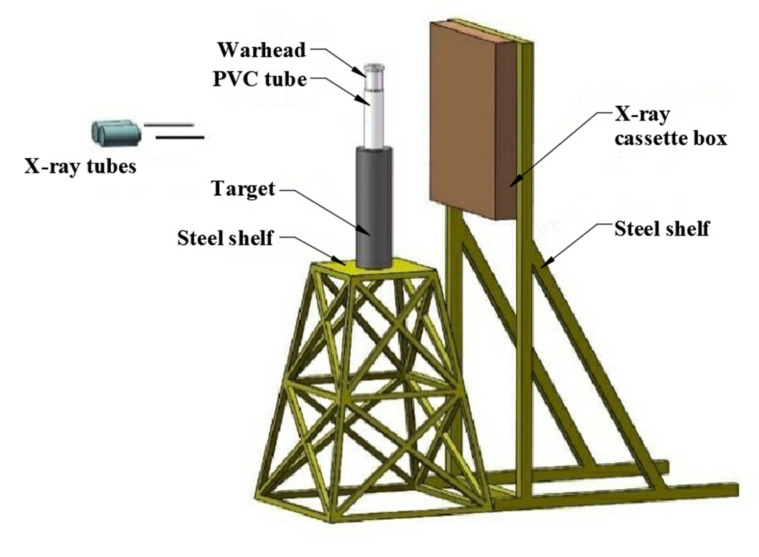
Schematic diagram of the experimental layout.

The jet performance tests included a jet appearance observation test and penetration test. A flash X-ray photography system (Scandish AB, at 450 kV) was applied, and the parallel photography mode was chosen. Therefore, the jet appearance at two different moments could be recorded on the same film for comparison. The timer was initiated after the detonation of the main charge using the synchronization control system. The assembled warhead was mounted right above the center of the target with a 200 mm PVC tube as the support, which is considered the optimum stand-off distance for the present test [26]. Two steel balls were attached to the PVC tube to calibrate the measuring. The targets used were stacked cylindrical 45# steel, with a diameter of 120 mm. The fabrication process of 45# steel is in accordance with standard [27] and the mechanical properties are listed in Table 2. The X-ray film was protected by the cassette box, which was fixed by the steel shelf at the opposite side of the X-ray tube. The schematic diagram of the experimental layout is displayed in Figure 7. The penetrated targets were retrieved and cleaned. A wire cutting process was used to cut the target longitudinally along the penetration tunnel to evaluate the jet penetration capability.

### 3.3. Micro Analysis of the Retrieved Targets

**Figure 8:**
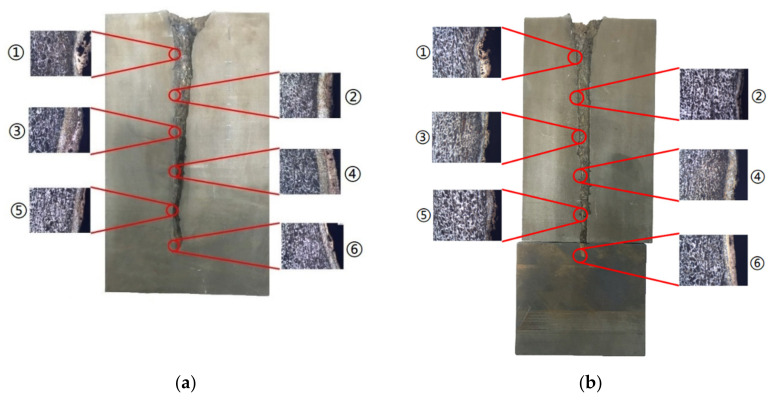
Segments selected along the penetration tunnel from the targets penetrated by (**a**) jet formed by turned liner and (**b**) jet formed by SLM-produced liner.

An in-depth analysis is required to offer a more specific explanation of the factors that influence the jet penetration capability. Thus, the micro view of the sidewall along the penetration tunnel was studied in order to compare the initial stage, middle stage and final stage of the jet penetration process. Considering the penetration depth caused by the two jets are different, segments for micro study can no longer be selected at an equal position on the penetration tunnel. Instead, six segments were selected at 1/6, 1/3, 1/2, 2/3, 5/6 and the end of each penetration tunnel separately to reveal the target micro performances during each corresponding penetration stage of the different jets. The selected positions on different targets were numbered ① to ⑥ as shown in Figure 8. The details of these selected positions will be analyzed in Section 4.4.

Each segment was cut into a 10 mm × 10 mm × 10 mm sample. A solution composed of 100 mL alcohol and 4 mL nitric acid was used to etch the samples after the polishing process. A micro view of each segment was observed using MV3000 metallographic microscope. A Tescan VEGA 3 integrated system was adopted for the SEM analysis and the EDS surface scanning. Vickers micro hardness equipment was applied to measure the micro hardness of different phase zones on the segments. A standard diamond indenter was chosen at a 100 gf load for 15 s.

## 4. Results and Discussion

### 4.1. Liner Microstructure

**Figure 9:**
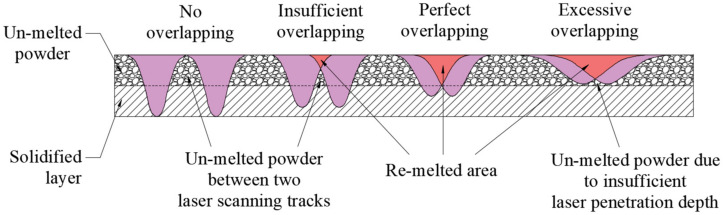
Schematic diagram of the overlapping area between two adjacent laser scanning tracks.

Micro views of the machined liner and SLM-produced liner are shown in Table 3. Strip defects appear on the longitudinal section of the SLM-produced liner. This defect is attributed to incomplete laser fusion. The overlapping area between the two adjacent laser scanning tracks is able to remove the keyhole generated by the temperature gradient along the material rise direction. However, the larger the overlapping area is, the more heat is dissipated around, and the heat penetration depth decreases, leading to incomplete fusion at the bottom of the overlapping area [28], as shown in Figure 9. Therefore, there is a higher possibility that the strip defect appears at the junction of two adjacent laser scanning tracks and this phenomenon is obviously seen on the longitudinal section of the SLM-produced liner. While as for the transversal section of the SLM-produced liner, no strip defect was obviously seen.

The grain sizes of these two liners are different. As revealed in Table 3, the grain size of the SLM-produced liner (around 15~50 μm) is much smaller than the machined liner, whose grain size lies between 200 μm and 600 μm. Considering the grain size difference between the two different liners and the clarity of the exhibition, a magnification of 100 and 200 times was chosen to illustrate the magnified view of the microstructures, as shown in Table 4. 

According to the report in [29], the grain of the SLM-produced material was found growing in a columnar way along the rise direction through several layers. However, in the present study, equiaxed grain was observed both on the longitudinal section and the transversal section and no columnar grain was seen obviously. Generally, heat treatment is applied to reduce the residual thermal stress and the heat treatment temperature will not trigger recrystallization. Yet, during the recrystallization process, the structure and properties of the material have undergone significant changes. The distortion-free equiaxed grains replace the original fractured and elongated grains. The strength and hardness of the material are reduced, but the plasticity and toughness are significantly improved. The heat treatment process in the present study not only reduces the residual stress inside the material, but also triggers grain recrystallization. Therefore, equiaxed grain was found both on the longitudinal direction and transversal direction. Additionally, the grain size of the material remains consistent with the original powder diameter. This phenomenon proves that SLM is another possible method of controlling liner grain size.

### 4.2. Jet Appearance

**Figure 10:**
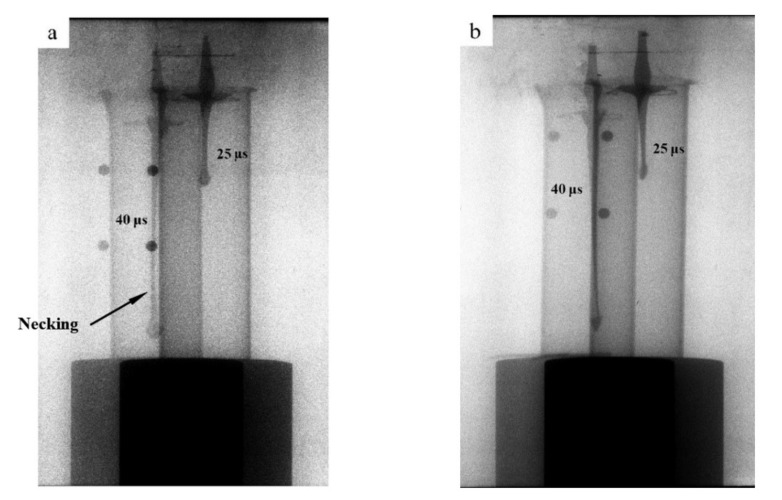
Appearances of jet formed by (**a**) turned liner and (**b**) SLM manufactured liner at 25 μs and 40 μs.

Driven by the detonated charge, liners were formed into jets. The jet appearances at 25 μs and 40 μs after the detonation were captured by the flash X-ray photography system, shown in Figure 10a,b. The tip velocities of the jets in Figure 10a,b were 6735.6 m/s and 6441.2 m/s, respectively, derived by dividing the distance between jet tip at 25 μs and 40 μs with the time interval. The tip velocity of the two jets should be considered the same, as the difference is only 5%. In Figure 10a, an obvious necking appears during the jet stretching process, while the jet in Figure 10b stretches continuously. The grain size is considered to be one of the factors that influences the jet stretching property. In 1995, Murr et al. observed the liner grain size before and after explosion loading. They found that different liner material has a different grain size ratio [7,8,9]. A relationship between the volumetric energy (*E*_0_) stored in the material and the starting grain size (*D_0_*) was proposed, as described in formula (1) [10]:

(1) $$E_{0}=(\Gamma \gamma_{gb}/D_{0})+(\alpha Gb^{2}\rho )$$

where Γ and α are material constants, $\gamma_{gb}$ is the specific grain boundary free energy, G is the shear modulus, b is the dislocation Burger’s vector, and ρ is the dislocation density. Besides, Murr suggested that the volumetric stored energy is the critical issue in controlling shaped charge jet stretching stability [10]. As the liner grain size becomes smaller, the volumetric energy stored in the liner increases, which improves the intrinsic driving force for plastic flow and energy minimization. During the jet stretching process, recovery and dynamic recrystallization occur [3,7,8]. This enables the material to have super-plasticity, which means the material can stretch under a large strain without necking [30]. Therefore, it is reasonable to believe that a finer grain size is beneficial for improving jet stretching performance, as proved in Figure 10a,b.

### 4.3. Penetration Capability

**Figure 11:**
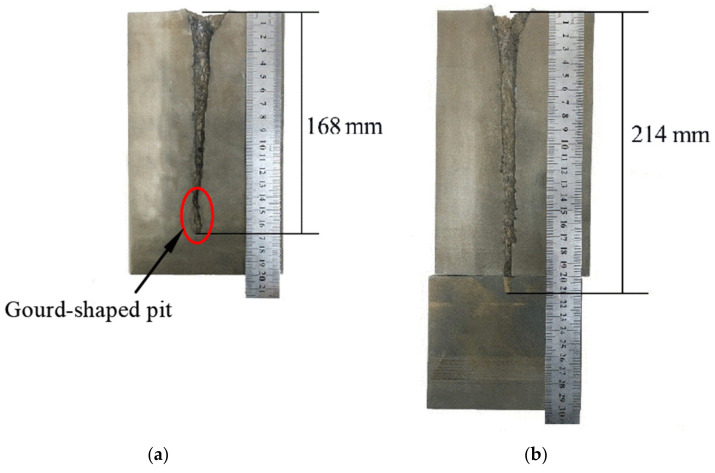
Penetration tunnels of jets formed by (**a**) turned liner and (**b**) SLM-produced liner.

The targets were retrieved and cleaned before cutting along the penetration tunnel and the penetration capabilities were evaluated. Figure 11a shows the penetration tunnel of the jet formed by the machined liner and the penetration tunnel in Figure 11b was formed by the jet generated by the SLM-produced liner. The penetration depth in Figure 11a is about 168 mm and the one in Figure 11b is 214 mm, which is 27% larger than the former. Similar results were also reported in [19]. A gourd-shaped pit is found at the end of the tunnel in Figure 11a. This is attributed to jet fracture during the stretching process.

During the jet stretching process, a fracture usually appears at the jet tip because of the large speed gradient [31]. When the jet scatters into fragments, the penetration process becomes intermittent. Typically, during the continuous penetration process, the jet energy is transmitted perpendicular and parallel to the penetration direction. The energy transmitted perpendicular to the penetration direction (or put briefly: laterally) increases the diameter of the penetration tunnel. The energy transmitted parallel to the penetration direction (or put briefly: vertically) increases the length of the penetration tunnel.

**Figure 12:**
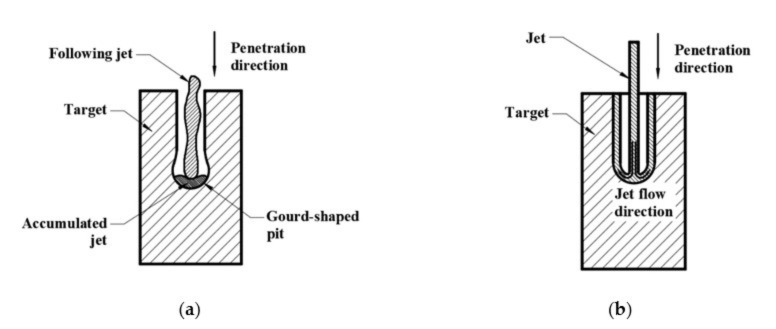
Schematic diagram of (**a**) intermittent penetration process with a gourd-shaped pit formed on the target and (**b**) continuous penetration process.

Figure 12a,b illustrate the penetration process of a fractured jet and a continuous jet, respectively. When the penetration process become intermittent, as shown in Figure 12a, the following jet fragments will first interact with the jet residue before interacting with the target. Therefore, the kinematic energy is transferred laterally, leading to an increase in the penetration tunnel diameter rather than its depth. As the following jet begins to accumulate, the vertical penetration process is hampered and the gourd-shaped pit forms, as shown in Figure 11a. Under such circumstances, the jet effective mass is reduced [4]. Consequently, the penetration process terminates due to severe jet erosion and a lack of penetration energy.

Figure 12b shows the penetration process of a continuous jet. After the interaction between jet and target, the jet residues stick to the sidewall and flow in an opposite direction to the penetration direction. The following jet is not hampered and interacts directly with the target, meaning that most of the jet energy is used to increase the penetration depth and less energy is transmitted laterally. The deeper penetration depth can be clearly seen in Figure 12b.

### 4.4. Microstructure Analysis of Target

**Figure 13:**
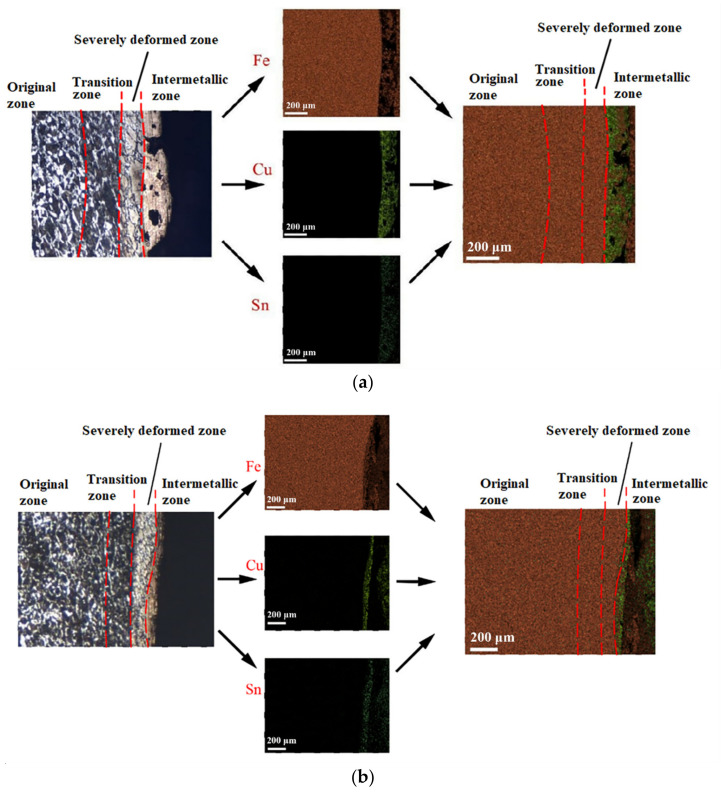
(**a**) EDS results of target penetrated by jet formed by machined liner. (**b**) EDS results of target penetrated by jet formed by SLM produced liner.

Micro views of the segments cut out from the penetration sidewall are listed in Table 5. The position number in the first column is consistent with that in Figure 8. It is clear that there are four different phase zones on each of the segments. The EDS surface scanning results are shown in Figure 13a,b, which indicates that the zone on the rightmost side of the metallographic graphs is composed of Fe, Cu and Sn. Thus, this zone is defined as the intermetallic zone (IZ). The zone on the left side of the IZ is defined as the severely deformed zone (SDZ), for the grain deformation is relatively severe. The zone on the left side of the SDZ is the transition zone (TZ), for the grain deformation level is lower than the SDZ, but higher than the original zone (OZ). The OZ is shown on the leftmost side of the metallographic graphs.

In Table 5, position ① is near to the entrance of the penetration tunnel, which experiences the early stage of the jet penetration. The intermetallic zone is generated by jet erosion during the interaction with the target. Jet residue was left on the sidewall of the penetration tunnel, which reduces the effective penetration mass of the jet. It can be seen from Table 5 that the width of the IZ is around 250 μm and it reaches its maximum at position ① both on the segments in column (a) and column (b). This phenomenon implies that the interaction between the jet and the target during the early stage of penetration is quite severe and the jet erosion is violent. At position ② to ④, the width of the IZ in column (a) maintains around 200 μm, while in column (b), the width is 20–70 μm. The difference between the width of the IZ shows that when a jet formed by the machined liner penetrates a target, severe jet erosion exists not only at the beginning of the penetration process, but also during the middle stage of penetration. As for the jet formed by the SLM-produced liner, the intensity of the jet erosion is lower during the middle stage of penetration. By comparing position ⑤ and ⑥, it is observed that the width of the IZ in column (a) is slightly smaller than in column (b). As analyzed in Section 4.3, during the final penetration stage of the jet formed by the machined liner, a gourd-shaped pit comes into being which causes jet accumulation. Therefore, the following jet does not interact with the target directly, and thus less material is found clinging to the sidewall. In the case of jet formed by the SLM-produced liner, the width of the IZ at position ⑥ becomes wider than at position ② to ⑤ in column (b). This phenomenon indicates that at the end of the penetration process, the jet was still interacting with the target rather than the accumulated jet.

**Figure 14:**
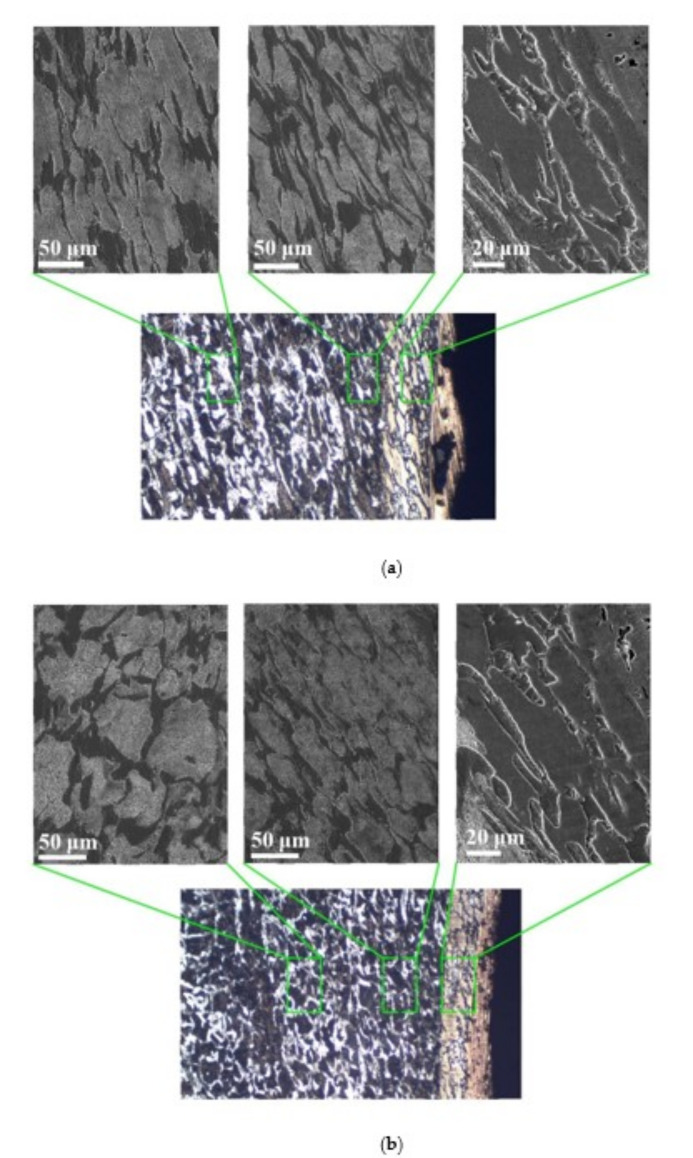
(**a**) SEM micrograph of tunnel penetrated by jet formed by machined liner. (**b**) SEM micrograph of tunnel penetrated by jet formed by SLM produced liner.

**Figure 15:**
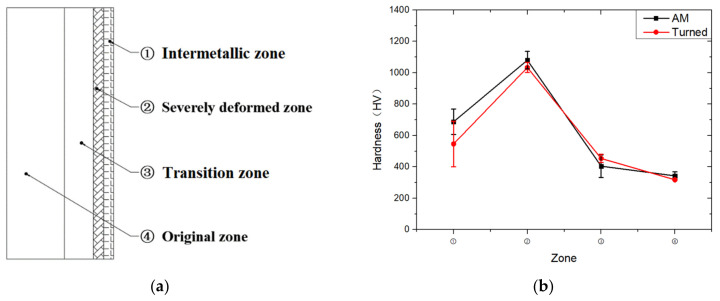
(**a**) Schematic diagram of four phase zones and (**b**) Micro hardness of each zone.

By comparing the EDS scanning results and the SEM micrographs, shown in Figure 14a,b, it is reasonable to believe that martensite forms in the SDZ. What is commonly known is that the formation of the martensite requires a huge amount of energy. For normal quenching martensite, the micro hardness value can only reach HV640-680 [32]. However, the Vickers micro hardness test results of the different zones on the segments selected, shown in Figure 15b, indicate that the micro hardness of the martensite generated on the sidewall of the penetration tunnel reaches over HV1000. According to [33], more than 90% of plastic deformation work transforms into heat under an extremely high strain rate situation and phase transformation initiates. This ultra-high hardness value proves the intense interaction between the jet and the target, which causes a large amount of lateral energy dissipation to form the martensites [21]. Thus, the width of the SDZ can be regarded as a clear indicator when evaluating the intensity of the lateral energy dissipation. As shown in Table 5, the widths of the SDZ in column (a) at position ① to ⑥ are larger than that in column (b), which suggests a large amount of lateral energy dissipation during the penetration process. According to the law of energy conservation, with the increase in laterally dissipated energy, the energy transmitted vertically decreases. Thus, the jet penetration capability is undermined.

As for the transition zone and original zone, less energy is transmitted to these two zones compared to the IZ and SDZ. Therefore, the micro hardness of these two zones is lower. Additionally, the laterally transmitted energy also causes grain distortion in the transmission zone. As seen in column (a) and (b), the TZ becomes narrower as the position gets nearer to the end of the penetration trajectory, which also implies that the intensity of the interaction between the jet and target decreases during the penetration process.

## 5. Conclusions

The SLM technique and classical turning methods were both used to fabricate shaped charge liners. The grain size of the SLM-produced liner was found to be much smaller than the machined liner due to the rapid heating and cooling rate during the SLM manufacturing process. Heat treatment after the SLM manufacturing process causes recrystallization and equiaxed grain was observed both on the longitudinal section and the transversal section of the SLM-produced liner. Besides, the grain size of the SLM fabricated liner keeps consistent with the original power diameter. This phenomenon suggests that the SLM technique is another possible way of controlling material grain size.

The jet forming performances of two different liners were observed. The tip velocity of the two different jets were almost the same, indicating that the influence of the manufacturing process on jet tip velocity is negligible. Necking appears at the jet tip formed by the machined liner, while the jet formed by the SLM-produced liner stretches continuously. Therefore, it is reasonable to believe that SLM technology is beneficial for improving the jet stretching stability.

More jet residue was observed clinging to the penetration tunnel penetrated by the jet generated from the machined liner. The composition of the segments selected was detected by energy dispersed spectrum (EDS) surface scanning. Four different phase zones were observed. SEM and Vickers micro hardness test results indicated that martensite appears in the severely deformed zone, which implies huge lateral energy dissipation that decreases the jet penetration capability. The width of the intermetallic zone and severely deformed zone reflects the severity of jet erosion and the intensity of the interaction between the jet and the target. The width of the intermetallic zone and severely deformed zone at six different positions on the tunnel penetrated by the jet generated from the SLM-produced liner reveals a continuous jet penetration process. During which, less residue jet is accumulated inside the penetration tunnel and left on the sidewall of the penetration tunnel. With less jet erosion and lateral energy dissipation, deeper penetration is achieved by the SLM manufactured shaped charge jet.

## Figures and Tables

**Table 1:** Chemical composition of CuSn10 powder.

Composition	Cu	Sn	Ni	Pb	Fe	Zn	O
Proportion	Balance	9–11	<0.01	<0.01	<0.01	<0.01	<0.2

**Table 2:** Mechanical properties of 45# steel [27].

Property	Yield Stress (MPa)	Ultimat Tensile Stress(MPa)	Elongation (%)	Section Shrinkage (%)
Value	355	600	16	40

**Table 3:** Metallographic micrograph of liners at 40 times magnification.

	Section	Longitudinal	Transversal
Technique			
Turning		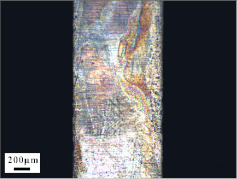	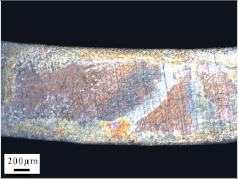
SLM		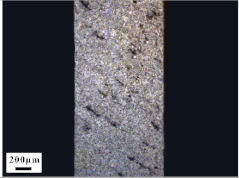	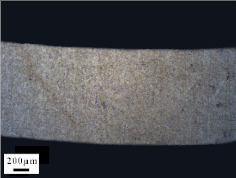

**Table 4:** Magnified metallographic micrograph of liners.

	Section	Longitudinal	Transversal
Technique			
Turning (100 times magnification)		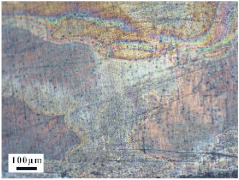	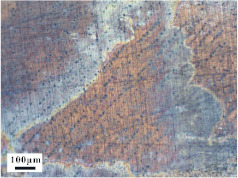
SLM (200 times magnification)		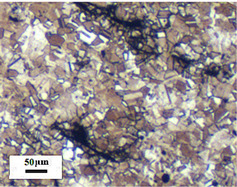	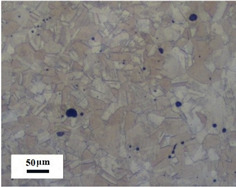

**Table 5:** Microstructure views of the sidewall on different targets penetrated by (**a**) machined shaped charge jet and (**b**) SLM-produced shaped charge jet.

Position	(a)	(b)
①	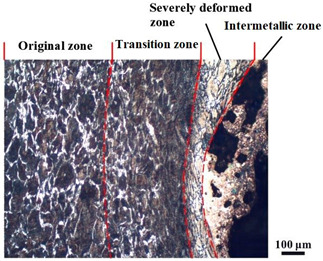	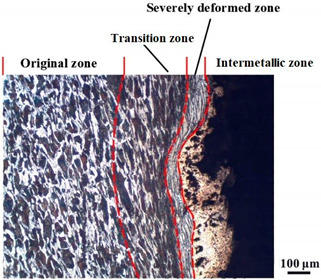
②	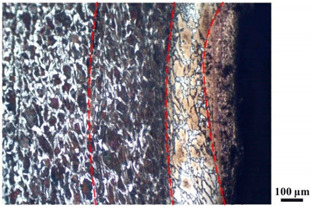	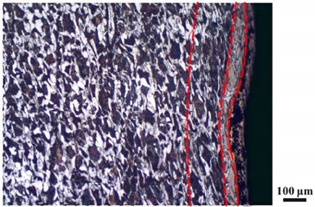
③	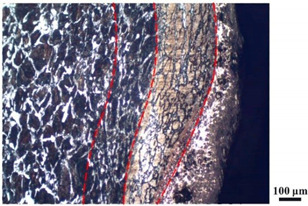	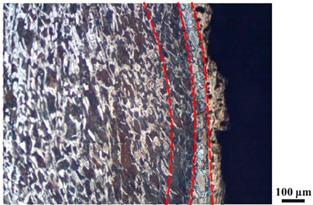
④	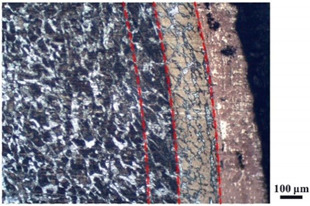	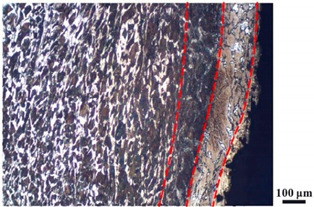
⑤	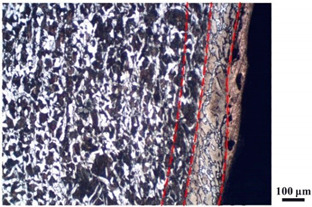	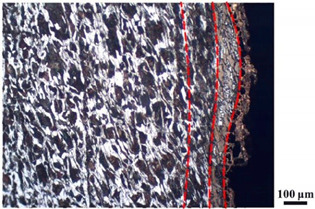
⑥	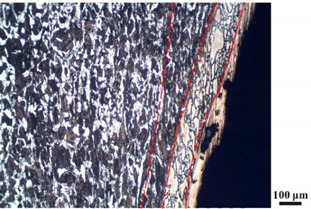	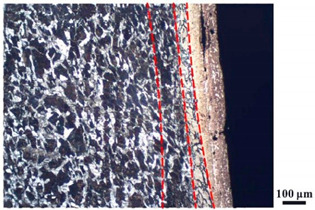

## Data Availability

Not applicable.
